# Bridging K-12 Student Mental Health Policy to Practice Gaps with a Multi-Component Framework

**DOI:** 10.1007/s10488-024-01396-w

**Published:** 2024-08-12

**Authors:** Lindsay Brindley, Paul Bauer, Alan J. Card, John Crocker, Nick Ialongo, Allen Tien

**Affiliations:** 1 Systems Improvement Facilitator, Eastern Upper Peninsula Intermediate School District, Sault Sainte Marie, MI USA; 2 Director of Systems Improvement and Evaluation Northwest Education Services, Traverse, MI USA; 3 Faculty in Global Health, The Health and Care Design Lab, San Diego, CA USA; 4 Department of Pediatrics, Division of Hospital Medicine, San Diego, CA 92123 USA; 5 Director of School Mental Health & Behavioral Services, Methuen Public Schools Founder & Director Massachusetts School Mental Health Consortium, Metheun, MA USA; 6 Director Center for Prevention and Early Intervention, Johns Hopkins University, Baltimore, MD USA; 7 President and Chief Science Officer Medical Decision Logic, Inc, Towson, MD USA

**Keywords:** Public mental health, Policy to practice gaps, K-12 schools, Multi-tiered system of supports (MTSS), Evolutionary systems improvement (ESI), BioPsychoSocialTechnical systems theory (BPST)

## Abstract

K-12 schools are a major sector for efforts to prevent and treat student mental health problems. In the United States, these efforts have led to the emergence of the MultiTiered System of Supports (MTSS) universal prevention, early intervention, and treatment policy framework. With a major focus on behavioral and mental health, MTSS has been adopted by all fifty state education departments. However, multi-level complexities of addressing student mental health within and across organizational structures complicate MTSS and broader policy development, implementation, and evaluation; disconnects between policy writers and practitioners obstruct progress, limiting positive outcomes. To bridge these policy-to-practice gaps, a multi-component solution is needed. The authors propose integrating the following elements: the Massachusetts School Mental Health Consortium’s Five Guiding Principles for Building a Coordinated School Mental Health System, the comprehensive school improvement methodology Evolutionary Systems Improvement (ESI); and the ontological framework of BioPsychoSocioTechnical Systems Theory (BPST). Individual application of these components has already yielded systems-level improvements outperforming compliance-driven procedures. Used together, these components offer a multi-level solution for establishing conceptually-guided, measurement-based loops that transcend the restrictions of uninformed policy, supporting stakeholders as they work to systematically eliminate barriers and improve student mental health.

## Public Mental Health and Education Interventions

Public mental health data indicate that rates of mental and behavioral health problems for children and youth have been increasing for decades, including harms and deaths from suicidality (Garnett et al., 2022), a situation exacerbated by COVID-19 and other emergent global problems (CDC, 2023b; Cimolai et al., 2021; Hay et al., 2021; Kampfschulte et al., 2021; Soberay et al., 2021). K-12 schools are a major sector for solving these problems, and have long been a focus for development of multi-level prevention interventions to improve mental health, evidenced by history and a growing body of scientific knowledge. (Fair et al., 2015; Kellam et al., 2011). Over the past several decades, emergent national organizations working in this area include the Collaborative for Academic, Social, and Emotional Learning (CASEL), the National Center for School Mental Health (NCSMH), the National Prevention Science Coalition (NPSC), and the National Alliance for Medicaid in Education (NAME), as well as the Massachusetts School Mental Health Consortium (MASMHC).

Broader education field efforts have focused on improving learning by identifying individual students with disabilities and intervening accordingly, such as the 2004 Individuals with Disabilities Education Improvement Act (IDEA) that promoted the measurement-based Response to Intervention (RTI) concept (Gresham et al., 2010), typically implemented with three categories: Tier (1) instructional content for all students; Tier (2) based on universal screening, supplemental group instruction for those students identified to be at risk of failure; and for those not responding, Tier (3) more intensive individualized intervention (Zhang et al., 2023). As the education community has increasingly recognized multiple aspects of developmental processes and capabilities for all students, the more descriptive term Multi-Tiered System of Supports (MTSS) has emerged and has developed a major focus on behavioral and mental health. However, although all 50 state education agencies (SEAs) use a tiered support model, there is large variation in naming and more importantly in assessment methods, interventions, use of implementation science, leadership, and management (Zhang et al., 2023), along with fidelity and scale of implementation. This variation may be one of the reasons that decades of work at multiple levels from research to policy to intervention has unfortunately not yet resulted in satisfactory population-level mental health.

### K-12 Student Mental Health Policy Development: Problem Patterns

About 10% of children and adolescents meet criteria for a mental health disorder, and far more are struggling with symptoms and risk factors (CDC, 2023a, 2023b). Accumulating evidence and bipartisan awareness of the scope of this mental health crisis has led to expansion of US Federal policies, programs, and funding to improve prevention, early intervention, and treatment within school systems. Examples include Substance Abuse and Mental Health Services Administration (SAMHSA), Garrett Lee Smith Suicide Prevention (GLS), Project AWARE (Advancing Wellness and Resiliency in Education), and School Based Trauma-Informed Support Services (TISS) programs. In parallel, state, county, and municipal governments are working to improve suicide prevention and to strengthen student mental health. Legislators have been active with new policies and programs, such as Michigan’s 31n (2018 law/funding to strengthen resources to improve student mental health)(MICHIGAN, 2018) and Maryland’s Blueprint (a ten-year plan launched in 2021 to improve K-12 education with a major behavioral health component, including community engagement) (Education, 2019). Consequently, state K-12 education and health and human services departments are increasingly tasked with school mental health policy and program implementation, working with Local Education Agencies (LEAs) and other community entities.

These policy developments, funding increases, and rising levels of participant activity motivate our efforts for this commentary, in which we seek to articulate key constraints on policy and program implementation and discuss underlying problem patterns in a manner useful to support more effective solutions. Our focus is on strengthening evidence-based practices, spanning development, dissemination, implementation, and integration. We suggest that better conceptualization of problem patterns that have hindered progress can increase confidence and capabilities for solving these problems. This understanding is important for problem-solving because, although the results of currently funded programs are yet to be fully observed, and are expected to help, the longitudinal trend of increasing mental health problem rates indicates that overall, existing policies and programs are still failing (CDC, 2023a, 2023b); this trend suggests that implementation of new policies and use of funds needed to be improved to prevent continued dysfunction.

Our perception is that most policy-program models lack a comprehensive and scientifically structured organizational, conceptual, and operational framework. A plethora of programs and projects distributed across multiple entities, e.g., school disttricts, generally leads to silos that are not well-connected and are often redundant. Policy makers work for years to develop and pass bills through labor-intensive legislative processes, but generally lack a standard measurement methodology for accurately monitoring, evaluating, and improving resultant program implementations (Card, 2022). Furthermore, the lack of a sufficiently comprehensive standard framework with which to aggregate and align individual policies means that each implementation is carried out with a mixture of previous practices, ad hoc reinvention, and partial problem solving, resulting in substantial variation in results. At the ground level, staff often lack confidence in their ability to innovate solution details, and local leaders struggle with validation concerns in choosing and carrying out new programs, overweighting the precautionary principle (Ricci & Sheng, 2013).

Overall, there appears to be insufficient conceptual and operational connectivity between policy specifications and what is required for successful practice. This lack perpetuates and may increase inefficiency, redundancy, and fragmentation, as well as causing missed opportunities to realize complementarities that could benefit students. Improvements in human structures and processes are needed to overcome prior performance deficits and reverse worsening trends.

## Conceptual Solution: A Three-Component Framework

As mentioned above, MTSS is rapidly emerging as critical to establishing a comprehensive school-based mental health system. It is visible as a common thread in the three components of our recommended solution which we believe provides core operational, conceptual, and organizational components while retaining the essential flexibility to meet the implementation needs of specific policies and programs. Recognizing the reality of systems inseparability, but also the utility of separation for discussion, three suggested components are as follows: (1) Five Guiding Principles for Building a Coordinated School Mental Health System (FGP): Guidance designed to support the emergence of measurement-informed prioritization and implementation of student mental health supports within a multi-tiered system framework. (2) BioPsychoSocioTechnical Systems Theory (BPST): A systems-based ontology framework for human-centered health improvement; (3) Evolutionary Systems Improvement (ESI): A comprehensive, adaptable school improvement methodology optimizing key aspects of a multi-tiered framework to support strategic thinking and systems design.

Early integration efforts suggest that, considered in tandem, these recently emergent components could provide leverage for scalable mental health systems improvement in schools and potentially more widely. By bringing together scientific models for addressing complex adaptive systems, continuous evaluation, and project operations to support and inform measurement-based policy adaptation and implementation, these components could be operationalized to overcome broader performance deficits and failures in K-12 mental health systems. Current knowledge suggests that using more direct measures of the processes that underlie causal mechanisms, the component parts of these processes and the interactions that lead to observed results, will increase effectiveness (Fath et al. 2019).

## Component 1: Five Guiding Principles for Building a Coordinated School Mental Health System (FGP)

The field of school mental health is increasingly acknowledged as an essential component of a prevention-oriented system of support for youth mental health (Lane et al., 2017). Despite this acknowledgment, significant barriers exist to implementing school-based mental health interventions without a sensitivity to the cross-sector nature of the field. School mental health occurs within an educational context yet interfaces and connects with community and public health contexts. For example, funding streams for mental health services are not universally directed toward the school context for provision of care or the development of systems of support. Rather, funding is often split between the two contexts of community *or* school (as opposed to community *and* school), resulting in siloed funding streams and inefficient policy and practice decisions. Without an explicitly defined set of shared responsibilities, support system implementation will remain convoluted, and implementation responsibility may fail to be claimed by any entity. A cross-sector solution that acknowledges the complexity of establishing policy and practice across contexts and defines the shared responsibility of multi-sector stakeholders is necessary to facilitate growth.

The Five Guiding Principles (FGP) for Building a Coordinated School Mental Health System were developed in conjunction with the growth and implementation of one such cross-sector effort: the Massachusetts School Mental Health Consortium (MASMHC). Building on the National Center for School Mental Health (NCSMH) model, approximately thirty Massachusetts school districts convened in January of 2018 with the expressed intent of developing a statewide learning collaborative focused on the development of Comprehensive School Mental Health Systems (CSMHS). NCSMH defines a CSMHS as “*school-district-community-family partnerships that provide a continuum of evidence-based mental health services to support students*,* families*,* and the school community*” (NCSMH, 2019). The articulated goal was to establish a statewide organizational structure unifying school systems focused on student mental health and standardizing the development and implementation of district-level CSMHSs across the Commonwealth. As MASMHC evolved, FGP emerged to provide influential considerations, conceptual underpinnings, and foundational methodologies.

When operationalized, these FGP can support the generation of measurement-informed prioritization rooted in prevention and reflecting an understanding of the organization of evidence-based practices (EBPs) within a multi-tiered system framework. These principles support a shared understanding of why students are receiving intervention, how they proceed through a series of supports based on data-driven indicators of need, and how the overarching system is nested within a larger and more complex system that will require consideration of the policies, procedures, and macro-level systems of systems that influence implementation. The following summarized descriptions of the Five Principles can be used to organize and prioritize implementation efforts while avoiding prescriptive “box checking” compliance, which unfortunately undergirds and overshapes many attempts at installing systems of support, often becoming a default mode of operation in the face of daunting complexity and perceived uncertainty and risk.

### FGP 1: K-12 Schools are the most Pivotal Prevention arm of the Mental Health System Writ Large

This guiding principle supports districts’ understanding of the responsibility placed on educational institutions to capitalize on their life-course duration and proximity to students, their ability to conduct aggregate assessments of the needs of populations (e.g. universal screening), and capacity to install systems of support that foster prevention and early intervention rather than the typical reactive stance (waiting to intervene in response to diagnosis and crisis alone). Rather than denoting a set of specific tasks associated with achieving this end, this guiding principle provides a starting point to inform reflection on implementation that is underscored by an opportunity, and a responsibility, to take action.

### FGP 2: MTSS Interventions can be Integrated to Support Students Comprehensively

This principle is based on the MTSS framework. MTSS encompasses services and supports offered across three categories of intervention at increasing levels of intensity (frequency, dosage, duration): Tier 1: universal prevention; Tier 2: topic-focused early intervention; Tier 3: indicated treatment. Addressing both mental health and school safety, MTSS could include Tier 4 crisis response. MASMHC and its partners support a comprehensive approach for implementation of MTSS, inclusive of a set of increasingly intense (in frequency, dosage, duration) interventions, as well as the requisite human systems infrastructure with which to deliver high-fidelity interventions. Districts must consider the etiology of the problem in question rather than simply react to the symptoms that are apparent and obvious, otherwise the elements of MTSS selected may not address the macro-level systems that influence outcomes. This approach increases the ability to go beyond the typically limited set of practices for eliciting small-systems change to a larger set of previously unconsidered, more comprehensive practices.

### FGP 3: School Mental Health Staff must Provide Evidence-Based Interventions and Supports

The cornerstone of quality implementation of a tiered system of supports is evidence-based practice. Adherence to practices proven through research to affect desired outcomes is requisite to the development of a quality system. This guiding principle orients implementers to consider a varied set of practices from which they can choose with their own discretion as opposed to a set of prescribed curricula that narrowly addresses disjointed issues. The evidence generated by the EBP can be used to ensure accurate selection and deselection of future interventions as well as supporting implementation of accurate and appropriately contextualized fidelity checks.

### FGP 4: External Partnerships are Helpful but not Sufficient

Similar to the call out to districts in FGP 1, this principle dispels perceptions that a “refer and hope” model of implementation (where districts simply refer a student to external mental health providers and hope the services help, recusing themselves from further responsibility) is sufficient. Districts grappling with the how of addressing student mental health needs can approach the resolution from many different angles. That being said, over-reliance on a community-based mental health system that orients primarily to diagnosis and crisis that is not designed to enact proactive and preventative needs identification and intervention in school settings, will prove inadequate to carry the weight of the youth mental health crisis.

### FGP 5: Clinical Leadership and Supervision Structures are Essential

Those responsible for evaluating the system and the staff therein should be knowledgeable experts in school mental health, decision-makers who are empowered to exercise their expertise and influence infrastructure. This design will allow the CSMHS to influence macro-level policies and procedures where feasible. Although many models of clinical supervision can be adopted, leadership structures are inherently required for optimal professional growth to be sustained. The absence of this guiding principle undermines the ability of school mental health staff to grow professionally; implement evidence-based practices; orient to the needs of students in a data-informed manner; and efficiently and effectively design and deploy services that meet the needs of the population they serve. Whatever cultural system has persisted over time will be adopted by those who populate it unless they are provided with leadership, direction, growth opportunities, and accountability markers for change.

As of 2024, MASMHC membership now exceeds 175 districts, and sponsorship of the consortium has grown to include institutions of higher education, regional mental health advocacy agencies, state agencies, and national organizations focused on the development of school mental health innovation. True to the BPST-supported commitment to participatory intervention design, member districts engage in resource sharing, development of best practices, and collaborative learning, leading to innovative implementation of the components of a CSMHS. Aggregated population data plays an important role in informing and guiding this process. Thousands of school mental health staff and educators have received training and resources from MASMHC; efforts to provide training, coaching, and technical assistance have advanced over time, allowing for deeper work within individual districts. Professional learning communities and coaching cohorts also receive advanced implementation support.

## Component 2: BioPsychoSocioTechnical Systems Theory (BPST)

In order to fully support the establishment of MTSS for mental health, systems-level complexities must be considered. When leveraging the FGP to drive CSMHS installation, we recommend users utilize BioPsychoSocioTechnical systems theory (BPST) to inform and increase accuracy of next steps.

The purpose of BPST is to extend the biopsychosocial model (BPS) and transform its structure by combining it with sociotechnical systems theory (Card, 2022). BPST offers a scientific structure for developing hypothesis-based studies that can generate new knowledge and more rigorous validations when compared to the original biopsychosocial (BPS) model (Engel, 1977). Although often referred to in public health and mental health discourse, including school-based mental health initiatives, the BPS model has generally failed to live up to its inspiring promise in terms of operational application (Bartholomew, 2023). This gap between *work as imagined* and *work as done* is a result of structural deficits in the BPS model. For one, the BPS model depicts determinants of health and disease (causes) and health status (effects) as a single nested system. Critically, it does not provide a testable process for changing this static understanding. The BPS model includes participant insights as an input for assessment and diagnosis, but not for bridging structural gaps with shared design, creation, execution, evaluation, and management of solutions.

**Fig. 1:**
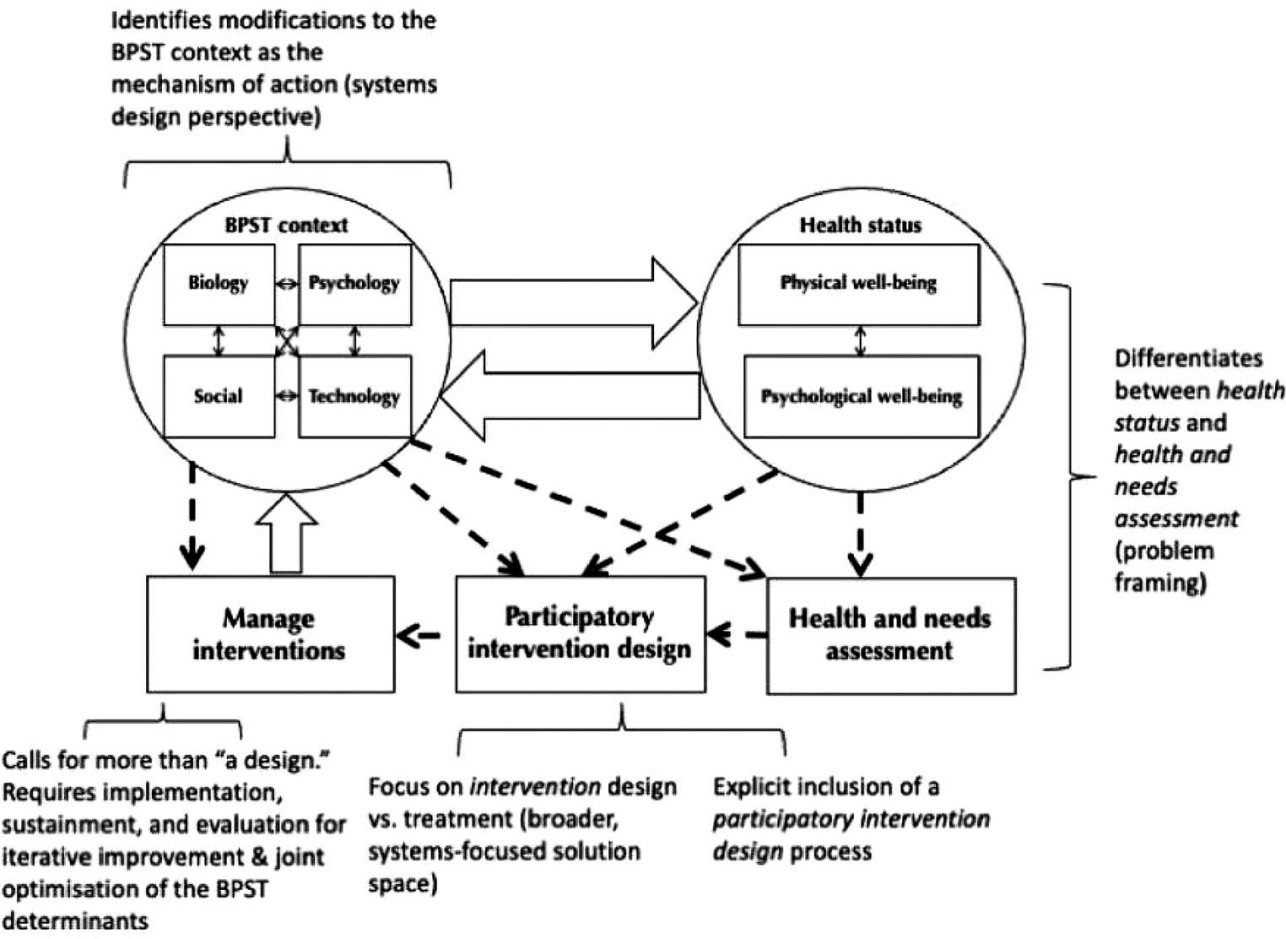
The BioPsychoSocioTechnical model (Card, 2022). Used under the terms of the creative commons attribution-noncommercial-noderivatives license

Developed to enable change in human systems, the BPST model conceives of a process for iterative integration of evaluation results into a larger, learning-based and sustainable process. In the past, districts struggled to prioritize MTSS-aligned practices associated with school mental health implementation as they worked to generate action plans. Driven by a commitment to facilitate continuous quality systems improvement, MASMHC has conducted statewide needs assessments to determine the most pressing areas of school mental health implementation for which districts require support and, in turn, resources, training, and coaching to directly address those needs in a tiered fashion. BPST states “*To purposefully rebalance the biopsychosociotechnical context*,* interventions must be implemented*,* sustained*,* and evaluated*” (Card, 2022). As a direct example of this principle, the power of the aligned state-level monitoring process of MASMHC is evident in the increasing accuracy of systems-level diagnoses associated with intervention provision. Implemented with additional specificity, BPST could further influence the definition of intervention design vs. treatment, where intervention design takes place in a broader, systems-focused solution space, shown below in Fig. 1.

## Component 3: Evolutionary Systems Improvement (ESI)

Effective improvement processes are integral to systems change. The structural framing of BSPT offers a general study design model for proposing and testing hypotheses generated through systems-level causation analysis and accurate gap identification. This function is operationalized in the newly emerging Evolutionary Systems Improvement (ESI) process. Recently developed to drive systems-level continuous improvement processes in education, ESI is a comprehensive, adaptable improvement methodology designed to support strategic thinking and systems design. ESI is specifically designed to address essential methods and processes for intervention in K-12 settings, using many of the same process components as BPST, adapted to negotiate the complexities permeating CSMHS installation in a multi-tiered setting.

Based in part on Human Performance Technology (Van Tiem et al., 2012) and incorporating the critical components of MTSS, ESI prompts close examination of current district-level processes and proficiencies, consistently inducing movement from inaccurate perception to accurate insight, and promoting this accuracy in systems–level solution selection. The broad evaluation criteria of ESI consist of three overarching drivers aligned with MTSS: (1) Data-Based Decision-Making; (2) Multi-Level Prevention; and (3) Screening and Progress Monitoring (AIR, 2024). Within each of these drivers exist five subdriver milestones for prioritization consideration (ex: Data System, Articulation of Teaching and Learning, Universal Screening); evaluative statements form an organizationally structured data flow to be utilized for prioritization conversations; MTSS-aligned solutions are defined within each subdriver. This design purposely integrates the perceptions of a variety of school personnel, providing varied access points and scaffolds to systems-level comprehension; true to BPST basics, it includes participant insights as “an input for assessment and diagnosis” and for “bridging structural gaps with shared design, creation, execution, evaluation, and management of solutions”.

ESI methodology is closely aligned with the improvement science principles supported by the Carnegie Foundation (Bryk et al., 2015). These principles include (1) Make the work problem-specific and user-centered; (2) Variation in performance is the core problem to address; (3) See the system that produces the current outcomes; (4) We cannot improve at scale what we cannot measure; (5) Anchor practice improvement in disciplined inquiry; and (6) Accelerate improvements through networked communities. These principles guide the collaborative routines experienced by ESI participants, providing both conceptual and concrete structures for the process.

Just as BPST’s meta-evaluative process centers on bringing tacit knowledge to the fore, ESI begins with an initiative inventory, including an integrated impact measure and the definition of evidence to support the identified impact (NIRN, 2020). Adhering to the principle of *seeing the system that produces the current outcomes*, this inventory process is designed to capture obvious initiatives in a district’s publicized curricular and instructional efforts, and the underlying decisions driving teachers’ day-to-day instructional framework. The thorough nature of this method authentically uncovers misalignments between what leadership assumes is happening and what is actually taking place with students each day, supporting the premise that *variation in performance is the core problem to address*, and addressing the gap between *work as imagined* and *work as done*, surfaced in the emgergence of BPST.

Early perception of the cause of poor performance is often tied to products; however, after further exploration, refinement of causal theories, and additional data gathering, users accurately ascertain that their performance issues are most closely tied to people and processes, making the work problem-specific and user-centered. To further cement this shift in thinking, initiative analysis formally incorporates a people/process/product (P3) data sort. The P3 model is not attributed to a single originator but has evolved over time within the education and instructional design field. The model underscores the importance of addressing all three components—people, process, and product—in designing effective educational experiences and promoting student success, supporting participants’ big-picture thought processes, and helping them understand how relationships between product-oriented implementation and process deficits can uncover inappropriate and even absent support of the human element of the system. This necessary understanding further solidifies the BPST call for feedback loops and directionality, rather than a siloed or atheoretical approach.

To enable teams to accomplish accurate causation identification and accurate solution selection, ESI facilitators support team repetition of the causation cycle, utilizing a series of questions designed to move participants from a typical entry point of tactics and logistics up the performance matrix (Gilbert, 2007). The causal theories utilized in these cycles are anchored in dominant leadership practices, as well as pivotal components of educational systems in general, making it possible to organize corresponding systems-level interventions under three headings: Leadership, Competency, and Organization (Fixsen et al., 2018).

This measurement-based identification is designed to reduce inefficiencies leading to process lag and eventual premature abandonment and to provide a reliable measure of progress, knowing we cannot improve at scale what we cannot measure.

## Discussion

While BPST states the importance of addressing intervention context is clear, currently, there is debate regarding if, when, and how to adapt interventions during local replications. While there is consensus that components deemed critical to success should not be altered, there is a growing concern that without an efficient strategy for updating evidence-based interventions (EBIs) and effective methods for adapting them to local circumstances, the population impact of EBIs might be reduced (Rotheram-Borus et al., 2012). The thirty-day cycles outlined in the ESI process allow for clarity around necessary adaptations, maintaining the integrity of the intervention at hand, and neutralizing questions regarding whether changes would diminish or enhance impact. It is important to note that responsive facilitation is not predicated on the improvement facilitator leading participants down a predetermined path, but rather using ESI as a thinking and acting framework that enables participants to proceed organically and authentically to the systems-level solution that uses measurement to reach the best impact on the complex system of systems that school mental health policies, programs, and people are part of.

Along with the propensity for accurate target identification taking hold, it is clear that the integration of fidelity considerations through ESI should be clearly articulated and increasingly leveraged. A survey of research revealed five main categories defining fidelity across sectors: Program Specificity, Adherence, Quality Delivery, Exposure/Duration, and Engagement (O’Donnell, 2008). When fidelity results for each area (high to low) were matched for analysis with outcome results (good to poor), inferences could be drawn from the paired data. These inferences could be used to influence the development and monitoring of implementation outcome targets already placed on an improvement continuum (Monday, 2022), adding both breadth and depth to the prioritization process. Current implementation efforts continue to solidify appropriate placement of the fidelity facilitation sequence, including experimentation with recent onboards introducing the fidelity considerations as early as initial environmental scan.

Not be viewed as an exercise in compliance that participants can move through in a linear fashion, the Five Guiding Principles instead serve as deeply foundational guideposts, or well-rooted yet transplantable trees of practice that implementers will need to address. Districts should approach implementation with an appreciation for the multiple points of entry that exist; they should engage in authentic needs assessment utilizing pivotal datasets to generate a dynamic action plan. Without intentional sequencing, districts may presume that a missing data source is the obvious starting point; they may invest valuable time and energy into development of a system that may only confirm a lack of need in that area. Of worse potential consequence is the assumption that a lack of data is confirmation of a need to implement a set of practices designed to improve that lagging dataset, as though the lagging data is indicative of a systems level need that should serve as a focal point of implementation. False positives and inauthentic data reflection practices may gravely undermine prioritization of the components of practice that a district ultimately chooses to develop.

Currently, lack of staff readiness (e.g. accurately assessing current reality linked to systems-level gaps and causation, selecting appropriate EBPs, implementing EBPs with fidelity, monitoring student progress and intervention impact, determining adjustments to practice) constitutes one of the greatest challenges facing the field of school mental health. If schools are to serve as the prevention arm of the mental health system, it is necessary to champion and develop staffing models that position school mental health (SMH) staff to deliver evidence-based interventions across all tiers. Too often, SMH staff are relegated to inappropriate tasking that does not effectively leverage their expertise; even worse, they often do not receive the necessary conceptual education, professional training, tools, and ongoing guidance at both implementation and clinical services levels. The dearth of clinical systems leadership in schools impacts practitioners negatively and undermines the development of systems and structures that foster strong clinical practice. An investment in clinical systems leadership may yield greater utilization of current staffing. Although not the obvious first choice, the results may be much more impactful than simply adding staff to a system that is not ready to fully realize their potential.

## Conclusion

Based on the above analysis and concurrent cross-sector implementations, we suggest that if engaged participants maximize the FGP to focus energy and resources through comprehensive school mental health efforts, optimize the ontological framework of BPST as an intentional approach, and operationalize ESI to drive effective systems improvement, they could make significant steps bridging policy-to-practice gaps. Because mental health outcomes mediate academic growth and achievement, student mental health is increasingly a focus of K-12 improvement efforts. Strengthening the general systems improvement process through ESI facilitation will partially close the gap; using the FGP to move forward with broad efforts such as MASMHC, ensuring principled adherence to EBP implementation and tiered support structures will also help. Conceptualizing and considering both components through the BPST lens strengthens each, clarifying and organizing what we have learned about these processes individually. If we are to link efforts across the health and education sectors to eliminate barriers to provision of student mental health supports, we must have a framework, a method, and a model to follow, and these three components provide an integrated solution, drawing on the power of cross-sector implementation to effect true systems change.
